# Differences in Associated Factors of Sedentary Behavior by Diabetes Mellitus Status: A Nationwide Cross-Sectional Study

**DOI:** 10.3390/jcm12175453

**Published:** 2023-08-22

**Authors:** Dong Kee Jang, Hyung Seok Nam, Mina Park, Yeo Hyung Kim

**Affiliations:** 1 Department of Internal Medicine, Seoul Metropolitan Government Boramae Medical Center, Seoul National University College of Medicine, Seoul 07061, Republic of Korea; mapmap05@snu.ac.kr; 2 Department of Rehabilitation Medicine, Sheikh Khalifa Specialty Hospital, Ras al Khaimah 6365, United Arab Emirates; hyung.nam@sksh.ae; 3 Department of Rehabilitation Medicine, College of Medicine, The Catholic University of Korea, Seoul 06591, Republic of Korea; mmaabb@catholic.ac.kr

**Keywords:** alcohol drinking, cardiovascular diseases, diabetes mellitus, exercise, sedentary behavior

## Abstract

This study aimed to identify the lifestyle and comorbidity factors associated with sedentary behavior by diabetes mellitus (DM) status. A total of 17,832 participants aged ≥50 years from the Korea National Health and Nutrition Examination Survey were included. Factors associated with long sedentary time (LST, ≥420 min/day) in individuals with and without DM (non-DM) were assessed. Among individuals with DM, LST was independently associated with excessive alcohol drinking (OR, 1.34; 95% CI, 1.02–1.74) and cardiovascular disease (OR, 1.47; 95% CI, 1.16–1.85). In individuals without DM, cancer (OR, 1.24; 95% CI, 1.06–1.44) and past smoking (OR, 1.16; 95% CI, 1.01–1.35) were independently associated with LST. Obesity (DM: OR, 1.28; 95% CI, 1.05–1.54; non-DM: OR, 1.24; 95% CI, 1.11–1.37), insufficient aerobic exercise (DM: OR, 1.55; 95% CI, 1.30–1.84; non-DM: OR, 1.50; 95% CI, 1.37–1.63), current smoking (DM: OR, 1.51; 95% CI, 1.11–2.05; non-DM: OR, 1.23; 95% CI, 1.05–1.45), and arthritis (DM: OR, 1.28; 95% CI, 1.04–1.56; non-DM: OR, 1.15; 95% CI, 1.04–1.27) were consistently associated with LST regardless of DM status. To reduce sedentary behavior time, it is important to consider an individual’s diabetes status and adopt a personalized approach.

## 1. Introduction

Sedentary behavior refers to activities that involve sitting or reclining with low energy expenditure [1]. Accumulating evidence has suggested that sedentary behavior is a crucial risk factor for several health outcomes, including diabetes mellitus, all-cause and cardiovascular disease mortality, depression, and some cancers [2,3]. The association between increased sedentary behavior and adverse health outcomes is significant, even after adjusting for physical activity or exercise levels, making sedentary behavior an independent behavioral risk factor for poor health outcomes [4]. Since sedentary behavior and diabetes mellitus are not only associated but share a common pathophysiology with diabetes mellitus as a clustered metabolic risk, increased sedentary behavior in individuals with diabetes mellitus can have a synergistic detrimental effect on health outcomes. 

Diabetes mellitus is an unmodifiable factor; however, sedentary behavior is a factor that can be modified through interventions, making it clinically important [5,6]. Therefore, understanding the factors associated with prolonged sedentary time is crucial for reducing sedentary behavior in individuals with diabetes mellitus. Obesity and metabolic syndrome are well-established factors associated with sedentary behavior in patients with diabetes mellitus [7,8]. In addition, depressive and anxiety symptoms are reportedly related to sedentary time in individuals with diabetes mellitus [9]. An association between sedentary behavior and comorbidities or inflammatory markers has also been reported in individuals with diabetes mellitus [10,11]. Although several studies have reported a wide range of factors associated with sedentary behavior in the general population [12,13,14], to the best of our knowledge, similar studies in individuals with diabetes mellitus are lacking.

Moreover, there is currently a knowledge gap regarding factors (other than the well-known metabolic factors) associated with sedentary behavior in patients with diabetes mellitus. Among these diverse factors, lifestyle factors and comorbidities are particularly important, as they can be modified, prevented, or treated. Furthermore, by identifying the differences in factors associated with sedentary behavior between individuals with and without diabetes mellitus, it would be possible to establish a more tailored approach to reduce sedentary behavior in individuals with diabetes mellitus. Therefore, the present study aimed to investigate and compare lifestyle and comorbidity factors independently associated with sedentary behavior in individuals with and without diabetes mellitus aged 50 years or above using participants from a nationwide database. We hypothesized that lifestyle and comorbidity factors independently associated with long total sedentary time in adults aged 50 years and older may differ based on diabetes mellitus status. 

## 2. Materials and Methods

### 2.1. Study Design and Participants

The current cross-sectional study utilized data from the Korea National Health and Nutrition Examination Survey (KNHANES), which was conducted by the Korea Centers for Disease Control and Prevention (KCDC). Data from the 6th, 7th, and 8th cycles of the KNHANES conducted from 2014 to 2019 were used. The KNHANES has been conducted every three years since 1998 and conducted annually since 2007. The KNHANES is a government-designated survey that collects information on health behaviors, prevalence of chronic diseases, and food intake of the Korean population [15]. The survey consists of three parts: a health survey, physical examination, and nutrition survey. The health survey and physical examinations were conducted at specialized mobile examination centers, and the nutrition survey was conducted through direct visits to households. All participants provided informed consent before participating in the KNHANES [15].

Among 47,309 participants of the KNHANES, individuals aged 50 or above were 20,546. After excluding 2714 participants with missing data, data on 17,832 participants (7709 males and 10,123 females) were analyzed. The KCDC publicly releases anonymized data from the KNHANES on its website, ensuring that the data are de-identified. As this study utilized publicly available anonymized data, it was exempted from review by the Institutional Review Board of Uijeongbu St. Mary’s Hospital, Republic of Korea.

### 2.2. Variables

The KNHANES assesses the levels of physical activity, including sedentary behavior, using the validated Korean version of the Global Physical Activity Questionnaire (GPAQ) among participants aged 19 years or older [16,17,18]. To evaluate sedentary time, we used Question 16 of the GPAQ, which asks, “On a typical day, how much time do you usually spend sitting or reclining?” The participants were asked to include the time spent sitting or lying down at work, at home, while moving between places and sitting or lying down with friends. Examples of sedentary activities included sitting at a desk; sitting with friends; using a car, bus, or train; reading books; writing; playing cards; watching television; playing games (Nintendo, computer, and PlayStation); using the Internet; and listening to music. Long sedentary time (LST) was defined as daily total sedentary time ≥ 420 min [15], and short sedentary time (SST) was defined as daily total sedentary time < 420 min.

Considering the World Health Organization (WHO) guidelines on physical activity and sedentary behavior [19], aerobic exercise level was regarded as sufficient if the participant met one of the following criteria: (1) moderate-intensity activity for at least 2 h and 30 min per week, (2) vigorous-intensity activity for at least 1 h and 15 min per week, or (3) an equivalent combination of moderate- and vigorous-intensity activities per week. Participants who did not meet any of these criteria were classified as those who engaged in insufficient aerobic exercise. Participants who performed resistance exercises such as push-ups, sit-ups, dumbbells, kettlebells, and pull-ups for two or more days in the past week were categorized as those who engaged in sufficient resistance exercise, whereas those who performed resistance exercises for less than two days in the past week were categorized as those who engaged in insufficient resistance exercise [19].

Participants were considered to have diabetes mellitus if they met one of the following criteria: (1) a diagnosis of diabetes mellitus by a doctor, (2) taking glucose-lowering medications, (3) receiving insulin injections, (4) hemoglobin A1c level of 6.5% or higher, or (5) fasting blood glucose level of 126 mg/dL or higher. Individuals with a systolic blood pressure of 140 mmHg or higher, with a diastolic blood pressure of 90 mmHg or higher, or taking antihypertensive medications were classified as having hypertension. Participants who were diagnosed with chronic obstructive pulmonary disease or asthma by a doctor or who showed abnormal results on spirometry (forced expiratory volume in 1 s/forced vital capacity [FVC] of less than 0.7 or an FVC less than 80% of the predicted value) were considered to have chronic respiratory disease. Individuals diagnosed with stroke, myocardial infarction, or angina by a doctor were classified as having cardiovascular disease [20]. Cancer, arthritis (osteoarthritis or rheumatoid arthritis), and depression were determined based on previous diagnoses by a doctor. 

Body mass index (BMI) was calculated by dividing measured weight (kg) by the square of height (m^2^). Participants’ obesity status was categorized according to the WHO’s guidelines for the Asia-Pacific region [21] as follows: (1) underweight (BMI < 18.5 kg/m^2^), (2) normal weight (18.5 kg/m^2^ ≤ BMI < 23 kg/m^2^), (3) overweight (23 kg/m^2^ ≤ BMI < 25 kg/m^2^), and (4) obese (BMI ≥ 25 kg/m^2^). Education level was classified into two categories: high school graduate or higher (>9 years) and middle school or lower (≤9 years). Individuals who worked one or more hours to earn an income or who worked as an unpaid family worker for 18 or more hours during the past week were considered employed. Participants who were originally employed but currently on temporary leave were also considered employed. Individuals were classified as living alone or with others according to the number of household members surveyed. The recorded data included information on marital status (married or unmarried), household income quartiles (low, lower-middle, upper-middle, or high), place of residence (urban or rural), and smoking habits (non-smoker, past smoker, or current smoker). Considering the recommended alcohol intake for patients with diabetes mellitus [22], alcohol consumption exceeding 20 g/day for men and 10 g/day for women was classified as excessive drinking.

### 2.3. Statistical Analysis

The basic sampling framework of the KNHANES consisted of the most recent population and housing census data available at the time of the sampling design. Following a two-stage stratified cluster sampling design, a representative sample of the entire Korean population was obtained. The KNHANES data included weights calculated using the design weight calculation, non-response adjustment, post-stratification, and extreme weight trimming. Using these weights to analyze the KNHANES data makes it possible to adjust for inclusion errors, uneven sampling, and non-response errors. Therefore, the KNHANES data was analyzed using a complex sampling design and weights to ensure that the results were representative of the entire Korean population.

In the present study, sociodemographic, lifestyle, and comorbidity factors were compared according to sedentary behavior time using the complex-sample chi-square test after stratification of the participants by diabetes status. The weighted prevalence of participants with LST was compared according to diabetes status using complex-sample chi-square test. To identify the factors associated with LST in individuals with and without diabetes mellitus, multivariable-adjusted complex-sample logistic regression analyses were performed. Since multi-morbidity is common in individuals with diabetes, we additionally analyzed the association between the number of comorbidities and LST among those individuals. The number of comorbidities was calculated by counting the number of diseases, including diabetes, hypertension, cardiovascular disease, chronic respiratory disease, cancer, arthritis, and depression. All statistical analyses were performed using the sampling weights and complex sampling design of the KNHANES. Complex sample analyses were performed using SPSS version 24 (IBM/SPSS, Armonk, NY, USA). A *p* value < 0.05 was regarded as statistically significant.

## 3. Results

### 3.1. Participant Characteristics

**Figure 1:**
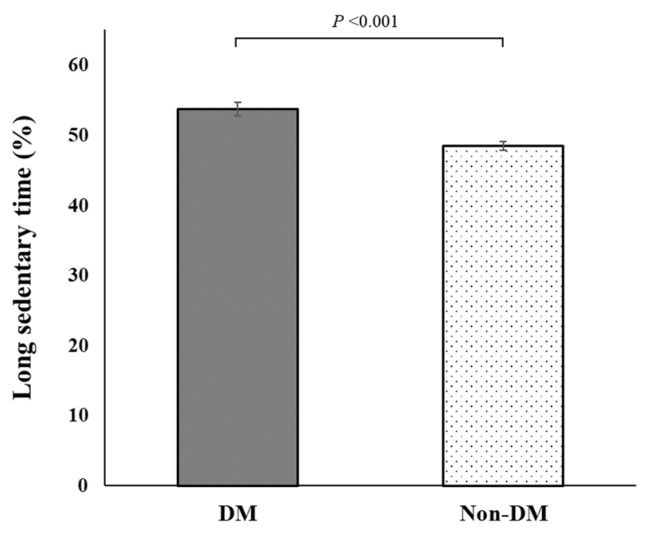
Weighted prevalence of individuals with long sedentary time by diabetes mellitus status. DM, participants with diabetes mellitus; non-DM, participants without diabetes mellitus.

**Figure 2:**
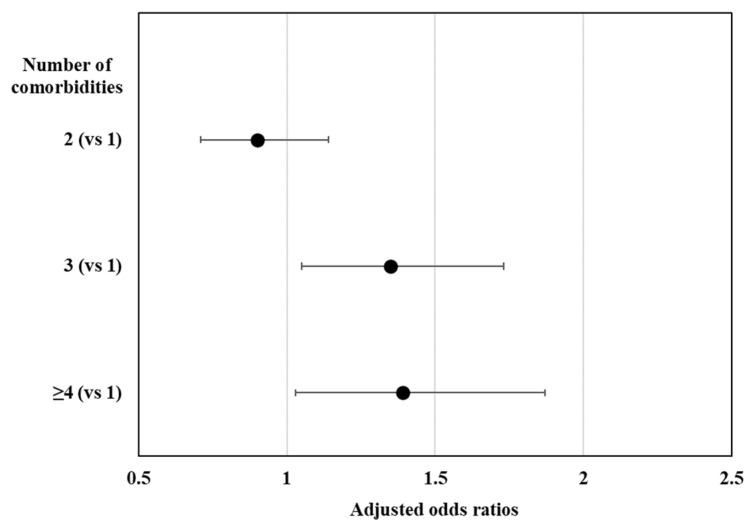
Association between the number of comorbidities and long sedentary time. Values are presented as odds ratios adjusted for age, sex, education, occupation, marital status, household composition, household income, residence, alcohol, smoking, aerobic exercise, resistance exercise, and obesity.

The characteristics of the participants with and without diabetes mellitus according to their sedentary behavior status are shown in Table 1. Among the 17,832 participants of the KNHANES aged 50 years or older, 3900 individuals had diabetes mellitus (unweighted prevalence, 21.87%). The weighted prevalence of diabetes mellitus was 20.64% (standard error [SE], 0.36). The unweighted prevalence of LST in participants with diabetes mellitus was 53.9% (2103/3900), and in those without diabetes mellitus was 48.6% (6777/13,932). As shown in Figure 1, the weighted prevalence of LST was significantly higher in individuals with diabetes mellitus (53.78%; SE, 0.97) than in those without diabetes mellitus (48.51%; SE, 0.60; *p* < 0.001). Figure 2 shows that the presence of 2 or more comorbidities was independently associated with LST among the participants with diabetes: ≥4 comorbidities (OR, 1.39; 95% CI, 1.03–1.87), 3 comorbidities (OR, 1.35; 95% CI, 1.05–1.73), and 2 comorbidities (OR, 0.90; 95% CI, 0.71–1.14) compared with 1 comorbidity (only diabetes).

As shown in Table 1, significant differences in some factors associated with LST, including older age, unemployment, living alone, low household income, urban residence, insufficient aerobic exercise, obesity, hypertension, cardiovascular disease, cancer, and arthritis, were commonly observed between participants with and without diabetes mellitus. In participants with diabetes mellitus, the percentages of females and participants with depression were significantly higher in participants with LST than in those with SST. A similar finding was not found among participants without diabetes mellitus. Differences in the proportions of education level and smoking habits by sedentary behavior status were significant in participants without diabetes mellitus but not in those with diabetes mellitus. The proportions of marital status, alcohol consumption, resistance exercise, and chronic respiratory disease did not differ according to the sedentary behavior status of participants with and without diabetes mellitus. 

### 3.2. Factors Associated with Sedentary Time by Diabetes Mellitus Status

Lifestyle and comorbidity factors associated with LST by diabetes mellitus status are shown in Table 2. Some variables showed a consistent positive association with LST, regardless of the presence of diabetes mellitus. Insufficient aerobic exercise was significantly associated with LST in participants with diabetes mellitus (OR, 1.55; 95% CI, 1.30–1.84) and without diabetes mellitus (OR, 1.50; 95% CI, 1.37–1.63). Current smoking was independently associated with LST in individuals with diabetes mellitus (OR, 1.51; 95% CI, 1.11–2.05) and without diabetes mellitus (OR, 1.23; 95% CI, 1.05–1.45). Obesity was independently associated with LST in participants with diabetes mellitus (OR, 1.28; 95% CI, 1.05–1.54) and without diabetes mellitus (OR, 1.24; 95% CI, 1.11–1.37). The association of arthritis with LST was significant in individuals with diabetes mellitus (OR, 1.28; 95% CI, 1.04–1.56) and without diabetes mellitus (OR, 1.15; 95% CI, 1.04–1.27).

Factors associated with LST in only the participants with diabetes mellitus were alcohol consumption (OR, 1.34; 95% CI, 1.02–1.74) and cardiovascular disease (OR, 1.47; 95% CI, 1.16–1.85), while those associated with LST in only the participants without diabetes mellitus were past smoking (OR, 1.16; 95% CI, 1.01–1.35) and history of cancer (OR, 1.24; 95% CI, 1.06–1.44).

## 4. Discussion

In the present study, several factors, including insufficient aerobic exercise, current smoking status, obesity, and arthritis, were independently associated with LST regardless of the diabetes mellitus status, while excessive alcohol consumption and cardiovascular disease were independently associated with LST in only the participants with diabetes mellitus. These results suggest that the lifestyle and comorbidity factors affecting sedentary behavior are different according to diabetes status in adults aged 50 years and older. 

This study found that excessive alcohol consumption and LST were independently and positively associated in individuals with diabetes mellitus but not in those without diabetes mellitus. This result is consistent with that of a previous report that unhealthy behaviors, including sedentary time, alcohol intake, and sugar-sweetened beverage consumption, co-occurred in clusters, and people who are obese had a higher chance of engaging in these cluster behaviors [23]. Furthermore, another longitudinal study indicated that excessive alcohol consumption was a predictor of sustained sedentary behavior in the adult population [24]. Although it is widely known that an unhealthy diet, excessive alcohol consumption, obesity, and sedentary lifestyle are risk factors for diabetes mellitus [25], previous studies did not adjust for diabetes mellitus status or perform stratified analyses by diabetes mellitus status. To our knowledge, the present study is the first to report an independent association between excessive alcohol consumption and sedentary behavior in individuals with diabetes mellitus. Alcohol consumption may be inversely associated with glycemic control, especially in patients with diabetes [26], and fluctuations in blood sugar levels may affect energy levels and physical activity patterns, potentially leading to prolonged sedentary behavior in these patients.

In the current study, a significant independent association was found between cardiovascular disease and LST in individuals with diabetes mellitus. A previous meta-analysis reported that prolonged sedentary time was associated with an increased risk of cardiovascular disease and cardiovascular and all-cause mortality [27]. Considering the results of the previous meta-analysis, the current stratified analyses by diabetes mellitus may weaken the association between sedentary behavior and cardiovascular disease in individuals without diabetes mellitus. Among individuals without diabetes mellitus, the initial significant association between cardiovascular disease and LST became insignificant after adjusting for multiple sociodemographic, lifestyle, and comorbidity factors. A sedentary lifestyle, which is a major risk factor for cardiovascular disease in individuals with diabetes mellitus, contributes to poor glycemic control [28]. Insulin resistance, often exacerbated by sedentary behavior, may promote the development of atherosclerosis and other cardiovascular complications [29]. Another explanation for our results can be inferred from recent studies showing that sociodemographic and lifestyle factors affect the association between sedentary behavior and cardiovascular diseases [30,31]. Additionally, there is a possibility that differences in physical activity or socioeconomic status between individuals with and without diabetes mellitus could have influenced the relationship between sedentary behavior and cardiovascular disease in our study [32]. 

Our study revealed that an independent association between obesity and sedentary behavior, regardless of diabetes mellitus status, was observed. Previous studies in various population and patient groups have consistently reported an association between obesity and sedentary behavior [8,33]. Our results in individuals with diabetes mellitus are consistent with earlier studies that reported an association between BMI and increased sedentary time among patients with diabetes mellitus [34,35]. Moreover, our findings in individuals without diabetes mellitus are similar to those of a previous study of Australian adults without diabetes mellitus that found significant associations of sedentary time with waist circumference and clustered metabolic risk, independent of their physical activity level [36].

In this study, a significant negative association was observed between aerobic exercise and sedentary behavior, but no association was observed between resistance exercise and sedentary behavior in individuals with and without diabetes mellitus. Therefore, individuals who were inadequately engaged in aerobic exercise were more likely to be sedentary than that observed in those who performed sufficient aerobic exercise, regardless of their diabetes mellitus status. The findings of previous studies, which indicate an inverse relationship between sedentary time and aerobic fitness, are consistent with the results of our study [37]. Among the various physical activity categories, such as aerobic exercise, strength exercise, flexibility training, and light walking, engaging in aerobic exercise is suggested to have behavioral and cardiorespiratory benefits [38]. Furthermore, a previous study found that engaging in aerobic or resistance exercise did not reduce non-exercise physical activity after the exercise [39].

The results of our study indicated that LST and current smoking were independently associated, regardless of the diabetes mellitus status. This result is consistent with those of a previous cross-sectional study in individuals with diabetes mellitus that reported current smoking as a risk factor for a high-risk physical activity profile, defined as high sedentary time (>648 min/day) combined with low moderate-to-vigorous physical activity (<30 min/day) [40]. Another study of the general population in the United States also found that sedentary behavior was higher in current smokers than in non-smokers and past smokers and that past smokers, compared with non-smokers, had higher sedentary behavior [41]. The independent association between sedentary behavior and current smoking suggests the possibility of a common pathophysiology between these two lifestyle factors. Smoking, dietary habits, and sedentary lifestyle are major modifiable risk factors for cardiovascular diseases, and they share common pathophysiologies such as endothelial dysfunction, smooth muscle dysregulation, and vascular inflammation [42]. In particular, in patients with diabetes mellitus, sedentary behavior, and smoking have been found to be independently associated with carotid plaques and atherosclerosis [43]. 

The current study found a significant positive association between arthritis and sedentary behavior independent of diabetes mellitus status. Our research findings contradict those of a previous study that reported similar sedentary behavior levels between older women with and without knee osteoarthritis [44]; however, the previous study did not adjust for potential confounders and included only older women. A recent study of individuals with rheumatoid arthritis suggested that disease activity indirectly influences sedentary behavior through pain intensity [45]. Nevertheless, to the best of our knowledge, studies on the association between arthritis and sedentary behavior are limited. Assessing physical activity levels in individuals with arthritis is challenging [46]. In addition, evidence regarding whether sedentary behavior worsens the outcomes of arthritis or whether increasing physical activity is beneficial is lacking [47]. 

The results of our study indicated an independent association between cancer and sedentary behavior in individuals without diabetes mellitus, but no independent association was found in individuals with diabetes mellitus. Previous studies have shown a cross-sectional association between cancer and sedentary behavior [48]. Furthermore, a longitudinal association between sedentary behavior and cancer occurrence has been consistently suggested; however, a clear relationship has not yet been established [49]. Considering the various cancer types and differences in their characteristics and survival rates, future research is needed to investigate the role of diabetes mellitus in the association between sedentary behavior and cancer.

The strength of the current study is the identification of lifestyle and comorbidity factors independently associated with prolonged sedentary behavior in a large cohort of individuals with and without diabetes mellitus and the ability to compare significant factors between the groups. Furthermore, this study adopted a complex sampling design for all the analyses. Therefore, the results can be interpreted as data from the entire Korean population. Nevertheless, this study has several limitations. First, the cross-sectional design of the KNHANES made it impossible to establish a causal relationship between the identified factors and prolonged sedentary behavior. Second, there is a possibility of recall bias because we collected data on some factors through surveys. Third, because this study used total daily sedentary time as a dependent variable, the results do not provide information on factors associated with domain-specific sedentary behavior. Furthermore, objectively measured sedentary time may be associated with factors other than that observed with subjectively measured sedentary time, as in the present study. Finally, because the present study was conducted in the Korean population aged 50 years and older, the results cannot be generalized to other age groups, races, ethnicities, or cultures.

## 5. Conclusions

Middle-aged or older individuals with diabetes mellitus who drink excessively or have cardiovascular disease, as well as those without diabetes mellitus who smoke or have cancer, are more likely to spend prolonged time being sedentary. Having diabetes mellitus did not show consistent associations with obesity, insufficient aerobic exercise, current smoking, arthritis, and LST in middle-aged or older adults. Therefore, to reduce sedentary behaviors, it is important to consider an individual’s diabetes status and adopt a tailored approach. Healthcare professionals should implement behavioral interventions, particularly regarding aerobic exercise, smoking, obesity, and arthritis, and focus on comorbidity control to reduce sedentary behaviors among middle-aged and older adults. Additionally, special attention should be paid to alcohol consumption behaviors and the presence of cardiovascular diseases in individuals with diabetes mellitus. Future prospective studies are needed to determine the causal relationships between the identified lifestyle and comorbidity factors in this study and sedentary behavior.

## Figures and Tables

**Table 1:** Characteristics of participants according to sedentary behavior time by diabetes mellitus status.

Variables	DM (n = 3900)			Non-DM (n = 13,932)		
	Short ST	Long ST	*p* Value	Short ST	Long ST	*p* Value
Unweighted number (n)	1797	2103		7155	6777	
Weighted number (n)	1,578,182	1,836,382		6,760,736	6,370,523	
Duration of diabetes (years)	6.94 ± 0.23	8.19 ± 0.22	<0.001			
Age groups			<0.001			<0.001
50–59 years	35.06 (1.41)	29.56 (1.22)		52.46 (0.77)	47.58 (0.85)	
60–69 years	37.54 (1.32)	30.28 (1.15)		30.95 (0.65)	26.22 (0.67)	
70–79 years	23.51 (1.07)	29.23 (1.07)		13.62 (0.46)	18.44 (0.54)	
≥80 years	3.89 (0.46)	10.93 (0.77)		2.97 (0.22)	7.76 (0.40)	
Sex			0.007			0.100
Men	56.00 (1.32)	50.90 (1.27)		44.81 (0.61)	46.41 (0.65)	
Women	44.00 (1.32)	49.10 (1.27)		55.19 (0.61)	53.59 (0.65)	
Education			0.826			<0.001
≤9 years	58.04 (1.48)	58.47 (1.32)		48.64 (0.87)	43.51 (0.91)	
>9 years	41.96 (1.48)	41.53 (1.32)		51.36 (0.87)	56.49 (0.91)	
Occupation			<0.001			<0.001
Employed	59.25 (1.36)	42.14 (1.27)		63.06 (0.72)	53.57 (0.78)	
Unemployed	40.75 (1.36)	57.86 (1.27)		36.94 (0.72)	46.43 (0.78)	
Marital status			0.448			0.144
Married	98.37 (0.36)	97.94 (0.41)		98.57 (0.17)	98.21 (0.18)	
Unmarried	1.63 (0.36)	2.06 (0.41)		1.43 (0.17)	1.79 (0.18)	
Household composition			0.025			<0.001
Living with other	85.41 (0.93)	82.52 (0.96)		89.99 (0.43)	86.85 (0.53)	
Living alone	14.59 (0.93)	17.48 (0.96)		10.01 (0.43)	13.15 (0.53)	
Household Income			0.004			<0.001
Low	29.59 (1.33)	35.86 (1.37)		20.47 (0.66)	23.79 (0.74)	
Lower-middle	27.49 (1.24)	25.62 (1.15)		26.96 (0.72)	22.93 (0.72)	
Upper-middle	22.64 (1.26)	18.81 (1.03)		26.40 (0.71)	22.53 (0.72)	
High	20.29 (1.24)	19.71 (1.14)		26.16 (0.83)	30.75 (0.92)	
Residence			<0.001			<0.001
Rural	25.45 (1.96)	17.82 (1.47)		22.16 (1.42)	16.30 (1.20)	
Urban	74.55 (1.96)	82.18 (1.47)		77.84 (1.42)	83.70 (1.20)	
Alcohol			0.599			0.789
Non-excessive	86.58 (0.98)	85.80 (1.02)		87.45 (0.49)	87.63 (0.49)	
Excessive	13.42 (0.98)	14.20 (1.02)		12.55 (0.49)	12.37 (0.49)	
Smoking			0.660			0.012
Never	51.17 (1.36)	51.99 (1.27)		60.49 (0.65)	57.43 (0.68)	
Past	30.72 (1.25)	29.16 (1.21)		24.36 (0.58)	26.51 (0.63)	
Current	18.10 (1.11)	18.85 (1.11)		15.16 (0.55)	16.06 (0.57)	
Aerobic exercise			<0.001			<0.001
Sufficient	41.80 (1.38)	30.94 (1.20)		46.27 (0.77)	37.15 (0.73)	
Insufficient	58.20 (1.38)	69.06 (1.20)		53.73 (0.77)	62.85 (0.73)	
Resistance exercise			0.378			0.143
Sufficient	18.04 (1.15)	16.75 (0.98)		21.61 (0.61)	20.40 (0.59)	
Insufficient	81.96 (1.15)	83.25 (0.98)		78.39 (0.61)	79.60 (0.59)	
Obesity			0.037			0.003
Underweight	0.92 (0.24)	1.08 (0.27)		2.47 (0.21)	2.96 (0.23)	
Normal weight	27.43 (1.25)	24.95 (1.09)		38.12 (0.71)	35.62 (0.69)	
Overweight	26.70 (1.17)	23.80 (1.13)		26.85 (0.64)	25.84 (0.64)	
Obese	44.94 (1.34)	50.17 (1.28)		32.56 (0.68)	35.58 (0.72)	
Presence of comorbidities						
Hypertension	59.82 (1.32)	66.57 (1.27)	<0.001	39.73 (0.69)	43.67 (0.75)	<0.001
Cardiovascular disease	10.11 (0.78)	16.12 (0.90)	<0.001	5.32 (0.31)	7.42 (0.36)	<0.001
Chronic respiratory disease	29.82 (1.31)	29.08 (1.17)	0.669	22.53 (0.59)	23.14 (0.61)	0.461
Cancer	7.37 (0.65)	9.41 (0.78)	0.044	6.67 (0.34)	8.34 (0.39)	0.001
Arthritis	19.64 (1.13)	26.39 (1.15)	<0.001	17.78 (0.50)	20.91 (0.55)	<0.001
Depression	4.91 (0.57)	6.73 (0.62)	0.033	4.99 (0.28)	5.78 (0.34)	0.061

Data are presented as means ± standard errors or percentages (standard errors), as appropriate. DM, participants with diabetes mellitus; non-DM, participants without diabetes mellitus; Short ST, participants with short sedentary time (<420 min/day); Long ST, participants with long sedentary time (≥420 min/day).

**Table 2:** Lifestyle and comorbidity factors associated with long sedentary time by diabetes mellitus status.

Variables	DM (n = 3900)		Non-DM (n = 13,932)	
	Unadjusted OR	Adjusted OR *	Unadjusted OR	Adjusted OR *
Alcohol				
Non-excessive	Reference	Reference	Reference	Reference
Excessive	1.07 (0.84–1.36)	1.34 (1.02–1.74)	0.98 (0.87–1.11)	1.02 (0.89–1.16)
Smoking				
Never	Reference	Reference	Reference	Reference
Past	0.93 (0.79–1.10)	1.21 (0.91–1.59)	1.15 (1.04–1.26)	1.16 (1.01–1.35)
Current	1.02 (0.83–−1.27)	1.51 (1.11–2.05)	1.12 (0.99–1.26)	1.23 (1.05–1.45)
Aerobic exercise				
Sufficient	Reference	Reference	Reference	Reference
Insufficient	1.60 (1.37–1.88)	1.55 (1.30–1.84)	1.46 (1.35–1.58)	1.50 (1.37–1.63)
Resistance exercise				
Sufficient	Reference	Reference	Reference	Reference
Insufficient	1.09 (0.90–1.34)	0.91 (0.73–1.12)	1.08 (0.98–1.19)	1.05 (0.94–1.17)
Obesity				
Underweight	1.29 (0.62–2.68)	1.13 (0.51–2.50)	1.28 (1.01–1.62)	1.10 (0.86–1.42)
Normal weight	Reference	Reference	Reference	Reference
Overweight	0.98 (0.80–1.21)	1.01 (0.82–1.26)	1.03 (0.93–1.14)	1.08 (0.97–1.19)
Obese	1.23 (1.03–1.46)	1.28 (1.05–1.54)	1.17 (1.06–1.29)	1.24 (1.11–1.37)
Presence of comorbidities				
Hypertension	1.34 (1.15–1.56)	0.93 (0.78–−1.10)	1.18 (1.09–1.27)	0.96 (0.88–1.04)
Cardiovascular disease	1.71 (1.38–2.11)	1.47 (1.16–1.85)	1.43 (1.22–1.67)	1.13 (0.96–1.34)
Chronic respiratory disease	0.96 (0.82–1.14)	0.95 (0.80–1.14)	1.04 (0.94–1.14)	0.98 (0.89–1.09)
Cancer	1.31 (1.01–1.69)	1.27 (0.97–1.67)	1.27 (1.10–1.47)	1.24 (1.06–1.44)
Arthritis	1.47 (1.23–1.75)	1.28 (1.04–1.56)	1.22 (1.12–1.34)	1.15 (1.04–1.27)
Depression	1.40 (1.03–1.91)	1.25 (0.90–1.73)	1.17 (0.99–1.37)	1.13 (0.95–1.34)

Data are presented as odds ratios (95% confidence intervals). DM, participants with diabetes mellitus; non-DM, participants without diabetes mellitus. * Adjusted for age, sex, education, employment, marital status, household composition, household income, residence, and all other variables in the first column.

## Data Availability

Data from the Korea National Health and Nutrition Examination Survey are available to the public on the website “https://knhanes.kdca.go.kr/knhanes/main.do (accessed on 28 May 2023)”.
